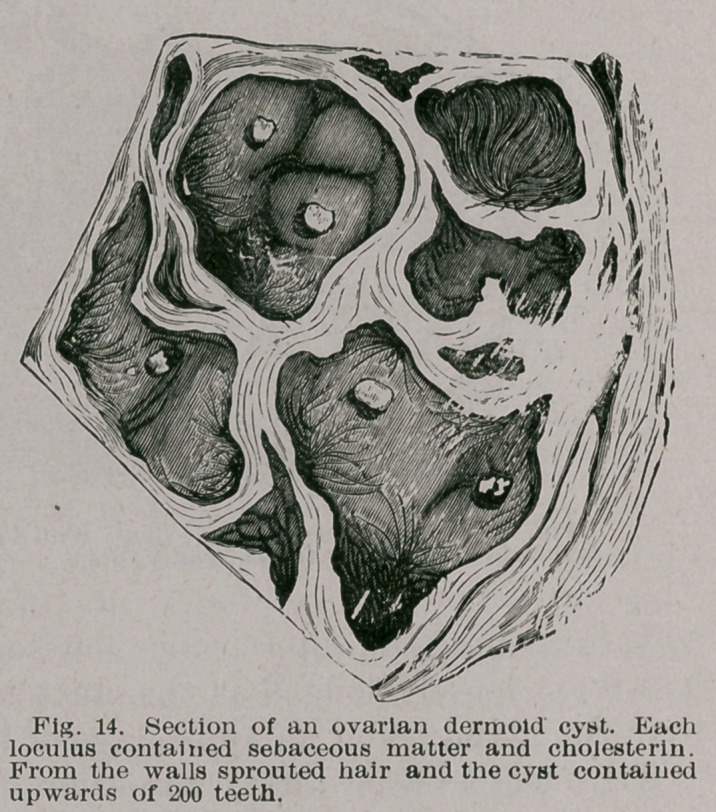# Teratomata—An Ætiological Study

**Published:** 1887-10

**Authors:** J. Bland Sutton

**Affiliations:** Hunterian Professor and Assistant Surgeon to the Middlesex Hospital. Erasmus Wilson, Lecturer Royal College of Surgeons, England


THE JOURNAL

OF

COMPARATIVE MEDICINE AND SURGERY.

VOL. VIII. OCTOBER, 1887. No. 4.

### ORIGINAL COMMUNICATIONS.

### Art. XXVIII. TERATOMATA. AN ÆTIOLOGICAL STUDY.

BY J. BLAND SUTTON, F. R. C. S.,

*Hunterian Professor and Assistant Surgeon to the Middlesex Hospital. Erasmus Wilson, Lecturer Royal College of Surgeons, England.*

The term *teratomata* in its most extended sense may be applied to those very remarkable congenital tumours which have always excited the interest of pathologists, not only from the variety of the tissues which enter into their composition, but from the most unexpected situations in which they occur. They may consist simply of skin possessing a few hairs and sebaceous glands, or they may contain in addition, bone, cartilage, muscle, fat, and even nerves. Although they exhibit a great tendency to occur in certain organs and regions of the body more frequently than others, nevertheless they enjoy a wide distribution.

The object of the present essay is to attempt a classification of this confessedly heterogeneous group by a careful consideration of their ætiology. The method adopted is to define the various classes and describe typical specimens in each class. For this purpose, although free use will be made of recorded cases, especial attention will be given to examples of those tumours I have personally dissected, or have had full opportunities of examining in museums or at the various London societies. This restriction of material is of pressing importance, for embryological and morphological researches have, during the last twenty years, brought to light many facts which will be freely utilized in the present study. As these facts were unknown to pathologists such as Morgagni, Lebert and Cruveilhier, the cases recorded by them cease to have much value, for the details in most instances are deficient in just those particulars which for the requirements of modern pathological anatomy would be of greatest value.

The cases recorded by these pathologists possess an interest, and are in a certain way important, inasmuch as they enable us to form tables whereby we may draw conclusions as to the relative frequency with which certain organs or regions of the body present such tumours.

We must now consider the definitions :—

*Teratomata constitute a class of congenital tumours composed of formed tissues derived from the epiblast and mesoblast, or from epi-meso and hypoblast.*

They form three groups, distinct not only in structural character but also in their ætiology :—

1. Dermoid cysts, arising from sequestrated portions of the epiblast.

2. Dermoid cysts and tumours arising in obsolete canals.

3. Parasitic fœtuses.

Before proceeding to deal with each group separately, it will be advisable to describe a typical example of each, as well as extreme forms in each division.

(1.) *Dermoid Cysts.*—The best examples of this group occur at the outer angle, less frequently at the inner angle of the orbit and at the root of the nose. The general appearance of a cyst at the outer angle is too well known to require a figure, but a drawing of the rarer variety is shown in Fig. 1. On opening such a cyst its interior will be found lined with skin; sprouting from the interior are hairs, many of which lie loose in the cavity mingled with epithelial scales, cholesterin and sebum, the latter being a product of the sebaceous glands which beset its walls. The hairs may resemble lanugo or the coarser hairs characteristic of the adult. Often the shed hair may be rolled up into balls as in fig. 2.

Fig. 1. A dermoid cyst at the inner angle of the orbit.

The hairs in a dermoid cyst may be two or three fine downy stems or innumerable. In length they are quite as variable, measuring in some cases a few lines, in others two feet. The colour is equally capricious and bears no relation to that on the body of the individual. In one reported case of a dermoid cyst occurring on the mesentery of a negress, blonde hairs were found. Different coloured hairs may be found in the same cyst, and a hair may be blonde at one extremity and black at the other. The colour of the hair changes with age. In animals the cyst will contain hair or wool according to the nature of the animal, and if in birds, feathers will be ig. 2. Dermoid Cyst from the back of a cow. (Museum Royal College of Surgeons.)

found.* In the majority of cases dermoid cysts are simple, but multilocular cysts occur with tolerable frequency. The most complex forms are found in the ovary.

Thus a dermoid cyst may be defined as a cavity lined by skin, furnished with dermal appendages, such as sudoriparous glands, hair with sebaceous glands, and in some situations, more especially the ovary, bone and teeth. No cyst can be termed dermoid unless it contains skin. On the other hand, all cysts containing skin are not necessarily dermoids, e. g., parasitic fœtuses. It is rare for "sequestration" cysts to possess teeth. These are usually found in those originating in obsolete canals, but are not confined to them.

Sudoriparous glands are not by any means always present in the walls of dermoid cysts, but occasionally they are present in vast quantities. In rare cases, examples of which will be referred to later, branched sebaceous glands are found, and in rarer instances a mammary gland. Commonly the skin lining the cyst wall differs from ordinary skin in having no horny layer.

(2). *Dermoids originating in obsolete canals.* This group includes those cysts which, like the preceding, are lined with skin, furnished with hairs and sebaceous glands. They may also contain teeth. Some dermoids belonging to this group may present themselves as tumours, the skin elements forming a capsule to the tumour, instead of forming the boundary walls of a cavity. The two forms agree in that they originate in obsolete canals.

We may select for our type lingual dermoids, often mistaken for ranulæ, but frequently described as sebaceous cysts of the tongue. Dermoid cysts of the tongue are retention cysts and arise in connection with the lingual duct, first described by His. This duct is represented in Fig 3. It is present in about ten per cent. of all human tongues examined. It runs from the foramen cæcum on the dorsum of the tongue to the posterior surface of the hyoid bone, where it ends blindly. In the ordinary course of events

*Lebert refers briefly to some examples of this nature.

the duct should suffer obliteration. Occasionally its position is indicated by an impervious fibrous cord. Should the extremities of this duct suffer obliteration and the intermediate part persist, the slow accumulation of epithelium and sebum distend it into a cyst. For a fuller discussion of the relation of this duct to lingual cysts, and also the connection of lingual dermoids situated between the anglo hyoid and genio-hyoglossus muscle, I would refer the reader to the reference given below*

Fig. 3. A longitudinal section through a human tongue, showing the lingual duct.

Dermoid cysts growing in the latter situation originate in abstricted portions of the branchial cleft usually known as the hyomandibular; they differ from cysts, originating the lingual duct only in the matter of situation, and like them are often confounded under the misleading clinical term, ranula. For dermoids arising in other situations of the neck from branchial spaces, see page 304.

In Fig. 4 is represented a rare form, known as a dermoid tumour. Tumours of this nature are solid and pedunculated. The exterior is covered with skin, from which hair grows, often many inches in length. The interior is made up of connective tissue, fœtal cartilage, fat, and sometimes nerve tissue. The most common seat for such tumours is the rectum. This form may possibly arise in the neurenteric canal, a passage whose nature and relation to dermoids will be fully discussed later.

Fig. 4. A dermoid tumour from the Rectum. (After Dr. Port.)

3. *Parasitic Fœtuses.*—The last group comprises those

*Ranulæ and Cysts in connection with the Hyoid bone. Path. Soc. Trans., Vol. XXXVIII., 1887.

extraordinary conditions known as parasitic fœtuses. They may vary from two fœtuses attached to each other by a narrow band, or they may be fused throughout the whole length of the trunk as in the amphibians in Fig. 5. The parasitic part may be simply a limb as in Fig. 6, or a shapeless mass growing from the sacrum, Fig. 7. In all these cases the deformity results from two fœtuses being developed from a single ovum. If both go on to full development a double monster is produced. If one part undergoes suppression, it simply becomes a shapeless, confused mass. This is known as the parasitic fœtus in contradistinction to the autosite or mature fœtus to which it is attached. In a case reported by Treves* as a congenital coccygeal tumour, the abnormal mass was found to be composed of skin possessing hair with developing sebaceous and sweat glands, masses of fat, cysts filled with mucoid material, portions of intestines, and bone covered with cartilage. Standing out from the growth were five nipple-like processes of flabby, hairless skin that resembled rudimentary digits. The largest of these exhibited vigorous contractile movements that were increased by stimulation with cold. Faint movements could, under certain conditions, be induced in the smaller processes. The mass was removed during life, and was found to be attached to the posterior surface of

Fig. 5. Two amphibians observed to be developed from one ovum. (Clark)†

Fig. 6. A parasitic embryo represented only by limbs. (After Tuckermann.)‡

*Path. Soc. Trans., Vol. XXXIII., p. 285.
†Anniversary Memoirs, Boston Soc. Nat. Hist., 1880.
‡Journal of Anat. and Physiology, Vol. XX., p. 576.

the coccyx and the lower half of the sacrum. Fig. 7.

Although parasitic fœtuses occur with greater frequency in the neighborhood of the sacrum than elsewhere, nevertheless they are by no means uncommon in other situations. The admirable Teratological collection in the museum of the Royal College of Surgeons possesses some excellent and remarkable specimens illustrative of the condition in such situations as the cranium, jaws, thorax, abdomen and the like.

Fig. 7. A parasitic fœtus removed from the sacral region of a child. (After Treves.) c, the largest digit. d, a piece of gut protruding through the slit, e. g, the pedicle.

For a fuller consideration of the facts relating to parasitic fœtuses, the reader is referred to a paper on the subject in the Gynæcological Journal, Vol. IX., p. 164.

Having sketched out the more important features of the three groups, we must now consider the evidence in favor of this mode of classification.

### SECTION I.—SEQUESTRATION CYSTS.

Dermoid cysts arising in detached or sequestrated portions of surface epiblast occur in the median line, and may be considered in three groups, I. DORSAL. II. VENTRAL. III. CRANIAL.

In order to comprehend in its full significance the ætiology of this interesting group, we must consider briefly the main features connected with the formation of the body walls. In a transverse section of the body of a mammalian embryo at an early period of gestation, we find that it consists of two bilateral portions united by an isthmus. This isthmus contains the notochord, flanked on each side by the mesoblastic somites. Its lateral parts present a smaller dorsal moiety which may be considered as upgrowths from the larger ventral portions. The dorsal upgrowths tend gradually to fuse across the median line to complete the spinal cord and the skin covering it. The tissue which serves to separate the cord from the integument, arises from the intrusion of mesoblastic elements to form the vertebral column.

Ventrally each half subdivides, the inner layer, the splanchnopleure eventually coalesces with its fellow to form the walls of the gut, the outer which is continuous at first with the amnion also fuses with its fellow to form the body walls, hence it is termed the somatopleure.

Coalescence may fail to occur at any spot along this extensive line or even throughout the whole length of it. If it fails at certain spots we may get such conditions as ectopia cordis and eventration.*

Dorsally this line of coalescence extends from the occipital protuberance to the coccyx, then passes beneath the notochord and extends ventrally involving the perineum, scrotum in the male, abdominal and thoracic walls, neck, symphysis pubis and finally becomes arrested at the septum nasi. The line of coalescence also extends from the septum nasi through the mid-line of the palate to the uvula. It also involves the tongue; the original cleft in this situation extends from the mental symphysis to the hyoid bone. In any part of this line epiblastic involutions occur, which may manifest themselves as dermoids. As is the case with any other species of tumour germs, three roads are open to these involutions. (1) They may disappear. (2) They may persist throughout life and cause no trouble, or (3) they may form cysts.

Examples of dermoids occurring in this line of coalescence will now be considered.

I. Dorsal Group.—Along the mid-dorsal line, dermoid tumours are very rare, and it is necessary to avoid confounding them with spina bifida tumours. The most typical case known to me is reported in the Journal de Medecine de Bordeaux, March, 1887. In this instance a dermoid

---

* Ectopia vesicæ admits of another explanation. See Shattock Path. Trans., 1887.

cyst occupied the middle line of the back. The interior was lined with skin furnished with hairs. The cyst was connected with the neural canal, and extended into the spinal cord so that its interior was in communication with the central canal of the cord.

This case is of exceptional interest when we reflect that in the early embryo the cells which ultimately constitute the epithelial lining of the central canal of the cord are directly continuous with those which are destined to become the superficial epithelium of the body.

II. Ventral Group.—*Scrotal Dermoids.*—There are very many good reasons for believing that the majority of dermoids reported as being testicular were really scrotal. This was clearly so in the following case reported by Mr. Bilton Pollard,* as a dermoid cyst of the testicle. It was situated in the left side of the scrotum between the testicles and was adherent to the back of the left one. It was outside the tunica vaginalis. The cyst was filled with a putty-like material in which there were a few grey hairs. Three grey hairs were found growing from the interior of the cyst-wall. The wall of the cyst was composed of fibrous tissue lined with stratified epithelium. The papillæ were very rudimentary; there were a few sebaceous glands in the cyst-wall.

Lannelongue gives reference to numerous cases in his work on Kystes Congenitaux, 1866.

*Penile Dermoids.*—The penis, as is well known, is formed by the union of two lateral moieties, the line of confluence being indicated in the adult by the prominent median raphé so conspicuous on the under aspect of the penis.

From what has been already mentioned regarding the predisposing circumstances leading to the formation of sequestration cysts, we may anticipate the occasional existence of dermoids in this situation.

Although penile dermoids are rare, nevertheless some instances have occurred. In one case referred to by Lannelongue, the cyst was connected with the prepuce. Other

---

* Path. Soc. Trans., Vol. XXXVII., p. 342.

authors have described small dermoids in a similar situation. It is rare to find them in any other part of the penile raphe, but an undoubted instance has been mentioned to me ; the cyst was attached to the penis, where it joins the scrotum.

The ventral line of coalescence extending from the penis to the cephalic end of the body is, like the corresponding dorsal region of the trunk, rarely the seat of dermoid cysts until we reach the angulus Luodvici, where they again become frequent.

An opportunity has never occurred to me of dissecting or even of examining a dermoid along any part of this line. Dermoids have been described as occurring at the umbilicus, but we must remember that ovarian dermoids exhibit a tendency, especially if they attain large dimensions, to become attached to the abdominal wall at this spot.

It must also be borne in mind that parasitic fœtuses are frequently found along this line and fœtuses attached by their sterna are of fairly common occurrence. Eliminating these cases, then we must come to the conclusion that the region from the manubrium sterni to the penis is a rare one for dermoid cysts.

*Dermoids in the mid-line of the neck.* Dermoids in this situation occur most frequently immediately above the manubrium sterni, at the spot known as the angulus Ludovici. In this spot they may attain goodly proportions and dip deeply behind the manubrium and invade the superior mediastinum.

It may be mentioned here that cases of imperfect coalescence along this line have been described by several observers. Among them may be mentioned Dzondi, Ascherson and Virchow. The defect consisting in minute canals, lined with epidermis capable of admitting a probe and leading to the trachea.

Dermoids have been described situated in the middle line between the symphysis menti and the hyoid bone, and Lannelongue* has figured and described a specimen of this nature which actually occupied the mental symphysis.

* Traite des Kystes Congenitaux; 1886.

The mode by which such cysts arise is well illustrated by a very rare specimen exhibited at the Odontological Society of Great Britain by Dr. Joseph Walker.*

It was the head of a calf which had a fissure of the palate running from the uvula to the premaxillary bone, but not involving the alveolus. The lower jaws with the skin covering them and the tongue were also cleft in twain, so that the symphysis could be widely separated. The cleft in the tongue extended to the basi-hyoid. The natural hairy integument, continued from the neck, passed on both sides of the cleft and extended quite up to the dorsum of the tongue. The specimen is one of extreme rarity and is of considerable interest; the defect is clearly an arrest of development, the tongue as we know being formed from two lateral portions.

*The Palatine Group.*—Dermoids occurring in the median line of the palate are of considerable interest, and tumour germs in the form of epithelial pearls have been known in this situation for a long time. It appears that neoplasms perfectly innocent in their nature, but full of epithelial nests occur in the palate, these take origin in little rounded masses of epiblast, inclosed between the two horizontal plates which fuse together in the median line in mammals in order to separate the nasal and buccal cavities. A good account of these epithelial involutions is furnished by Leboucq.†

Mr. Stephen Paget‡ has considerably advanced our knowledge of palatine tumours and has collected several examples of buccal dermoids. As a type case the following may be given:

"A male infant was born with a tumour inside its mouth which was at first mistaken for its tongue, being of the same color and consistence; the soft palate was partially cleft. From the middle of the hard palate grew a tumour, which was lobed, covered with silky hairs, especially on

---

* Vide Odontological Transactions, 1887, for a drawing of the head.

† St. Bartholomew's Hospital Reps., Vol. XXII., p. 315.

‡ "Le Canal naso palatine chez l'Homme" and Note sur lez Perles Epithelials de la Voute Palatine Archives des Biologie, Vol. II., 1881.

the left side. Its surface was of true skin, with large sebaceous glands, but no papillæ, containing vessels or nerve-filaments ; inside it was composed of adipose tissue with a central fascicle of striped muscular fibre radiating outwards towards the surface." *Clerault, Bull. Soc. Anat.* 1874, p. 380. All tumors comtaining hairs, occurring in the buccal cavity, are not necessarily dermoids, or even attached fœtuses. The stomodæum acquires its epithelium from the epiblast, and in some mammals, e.g., the hare and porcupine, the interior of the cheek is furnished with hair. Thus cutaneous tumours of the mouth may be dermoid, parasitic fœtuses, or atavistic in their nature.

The preceding group of cases deals with cysts arising by sequestration in the middle line of the body, dorsally and ventrally. Before proceeding to discuss those cysts which originate in obsolete canals, it will be well to consider an intermediate variety connected with the iris. Cysts variously described as sebaceous and dermoid, growing in the iris, may arise in two ways. The majority of cysts of this character, connected with the iris, have been associated with antecedent perforating wounds of the cornea. Clinical and experimental evidence support the view that small fragments of epidermis conjunctival epithelium, or eyelashes implanted accidentally or designedly upon the iris, may, and do, grow into cysts.

Mr. Osborn* suggests that some of these cysts may occasionally be due to the dilatation of some portion of the unobliterated remains of the choroidal cleft to the persistence of which the term coloboma iridis has been applied. They look like small white currants, semi-transparent and pearly, protruding themselves into the interior chamber, occluding the pupil and eventually inducing an attack of iritis.

Osborn also relates a case in confirmation of this view where there was not only a cyst of this kind on the iris, but also a dermoid in front of the ear a little above the tragus.

Further evidence has recently been published by M.

*St. Thomas Hosp. Rep., Vol. VI., p. 69, and R. Lond. Ophth. Hosp. Rep.. Vol VII., p. 245.

Masse* which supports Osborn's views. Cysts of the iris are of rare occurrence, and they are among the most striking and beautiful pathological specimens when seen in the living eye.

III. Cranial Group.—*Dermoids in connection with the Cranium.*—Dermoid cysts occur with tolerable frequency in connection with the cranium, and in certain definite positions. The most frequent situation is in the immediate vicinity of the external angle of the frontal bone, also fairly often at its internal angle and in the middle line of the forehead at the root of the nose. Other but less common situations are the external auditory meatus, and the pinna. Dermoids have been described as situated in the middle line of the skull, over the anterior fontanelle and in the tentorium in relation with the torcular Herophili.

In all these situations care is necessary in order not to confound such cysts with sebaceous cysts, and to be careful to discriminate between meningoceles and dermoids when the tumour is situated in the middle line of the skull.

An analysis of a large number of these cases collected from standard and periodical literature, as well as a careful examination of numerous cases I have observed and removed, has served to convince me that with the possible exception of those occurring in the external auditory meatus dermoids of the cranium belong to the sequestrated variety. The argument in favor of this view runs as follows: The external ear or pinna is formed by the coalescence of a series of tubercles, six in number. Very frequently these tubercles fail to fuse completely and a fistula lined with epidermis results. It is easy to see that if a space is left between two tubercles, without any external opening, its subsequent dilatation will bring it into the category of cysts.† Those dermoids occupying the tympanum probably arise in the same way as those discussed in the section devoted to branchial dermoids.

*See Kystes Tumeurs perlees et Tumeurs dermoides de l'Iris Paris, 1885.

† We are indebted to the careful work of His for a clear and accurate account of the development of the pinna. Its relation to congenital fistulæ of the pinna I have discussed in the Journal of Anat. and Phys., Vol. XX , p. 289.

The outer and inner angles of the orbit mark the points where there is every opportunity for a piece of the epidermis to become involuted, as it is the seat of a considerable fissure during embryonic life. It is stretching the imagination to regard defects in this position as originating in branchial fissures, for the anterior limit of these curious clefts is indicated by a line drawn from the angle of the mouth to the external auditory meatus.*

Dermoids occurring at the inner angle of the frontal bone must be divided into two classes: Those lying in relation with the orbit, and those situated at the root of the nose. The orbital variety clearly arises in the same manner as those described at the outer angle of the frontal bone.

The dermoids which remain for consideration are those at the root of the nose in the middle line of the cranium, and those lying on the dura mater. Those at the roof of the nose often project between the nasal bone, whilst others cause absorption of the frontal and are in contact with the dura mater. These simulate very closely pedunculated meningoceles. Dermoids occur over the anterior fontanelle and simulate meningoceles.†

Prof. Turner‡ has recorded a description of a cyst which lay within the layers of the dura mater, beside the torcular. It contained hair and was unassociated with any defect in the occipital bone. It occurred in a child twenty-three months old.

Dr. Ogle§ has observed a similar case in a child two and a half years old. It was associated with a defect in the squamous portion of the occipital bone.

Cysts of these characters in such situations receive a satisfactory explanation when we consider the development of the skull vault. Morphologically regarded, the bony framework of the skull is an additional element to the primitive cranium, which is really represented by the dura mater. Strictly, the term extra-cranial should apply to all structures outside the dura mater. As a matter of fact, in early

* See especially Cusset, *Sur l'appareil branchial des Vertebres.*
† Giralde's Lecons Cliniques, p. 342.
‡ St. Barth. Hosp. Rep., Vol. II., p. 62.
§ Brit. and For. Med. Chir. Review, 1865.

embryonic life the skin and dura mater are in contact. This relation later becomes disturbed by the intervention of the cranial bones. If an aberration of the skin occur before the advent of ossification, the detached portion may be pushed towards the dura mater, or the skin, or retain, by means of a pedicle, its old attachment to the latter, whilst the bulk of the cyst lies on the inner side of the bone.

Hence in cases of cranial dermoids surgeons often find the pedicle of a cyst leading through an aperture in the bone and connected with the dura mater. In my opinion the current explanation of this condition, viz., that the cyst by pressure brings about absorption of the bone, is in such instances incorrect. In the formation of the bones of the cranial vault, the epidermic covering of the primitive skull was not fully detached from the dura mater, and the bone actually formed around the pedicle.

This is well illustrated in the specimen from which Fig. 8

Fig. 8. A dermoid tumour over the parietal bone, connected by a fibrous pedicle with the dura mater. It contained sebaceous matter, cholesterin and hairs.

was taken. The parts are preserved in the Museum of Middlesex Hospital, and described as a sebaceous cyst which had produced absorption of the parietal bone ; it was connected with the dura mater by a band of fibrous tissue. As far as I am aware no instance of a sebaceous cyst has yet been recorded, connected with the dura mater ; this induced me to re-examine the specimen. Upon cutting into the cyst it was found filled with pultaceous matter mixed with a large quantity of light hairs.

### SECTION II. DERMOIDS ORIGINATING IN OBSOLETE CANALS.

By an obsolete canal is understood, in this article, a canal or duct which, in the ancestors of mammals was functional, but which re-appears in existing forms in obedience to the law of heredity. In the ordinary course of development these canals disappear; occasionally they remain persistent, and in some instances act as cyst-germs. The characteristics of these passages are these: they are lined with epithelium, they traverse a mass of mesoblastic tissue, and in many instances serve to bring the epiblast and hypoblast into temporary relation.

The more important of these obsolete canals are:—

1. The cranio-pharyngeal canal.
2. The neurenteric passage.
3. The lingual duct.
4. The branchial clefts.
5. The peritoneal funnels in relation with the ovary.

1. *The Cranio-pharyngeal Canal.*—This passage is a diverticulum from the stomodæum and is situated between the open arms of the trabeculæ cranii and comes into contact with the infundibular process of the anterior encephalic vesicle. The remains of this canal is often very clearly indicated in the adult by a recess in the mid-line of the pharynx, close to its roof, known as the bursa pharyngea, or pouch of Rathke.*

Some typical examples of dermoids arising in this region have been placed on record in the transactions of the London Pathological Society by Messrs. George Lawson, Hale, White and Bowlby. They may be closely associated with the body of the sphenoid bone, or hang as pedunculated pilose tumours into the pharynx.

---

*For all that relates to the development of these obsolete canals and their embryology, in so far as it bears on this subject, the reader should refer to my General Pathology, Chap. XX.

2. *Cysts of the Neurenteric Passage.* This remarkable canal continues the central canal of the spinal cord and passes round the caudal end of the notochord and opens into the post-anal gut, Fig. 9. An investigation into the fate of this canal leads one to believe that it is by dilatation of this passage that those dermoids arise which occupy the space between the rectum and sacrum. Should they attain large proportions they bulge upwards and occupy the pelvis. The dermoids which I believe to arise in connection with this duct, present very definite characters and manifest themselves in two forms—either as dermoid cysts, or the rarer variety of dermoid tumours. The cysts are usually situated on the posterior (dorsal) aspect of the rectum and may occur at any spot between the anus and sigmoid flexure. These cysts may contain skin, hair, sebaceous and sweat glands, teeth, bone or cartilage. A typical example is represented in Fig. 10.

Fig. 9. Longitudinal section of an embryo *Bombinator igneus*. A, anus; B, brain; N, notochord; M, mouth; L, liver; N. C., neurenteric canal; M. C., medullary canal (after Goette.)

Fig. 10. The posterior (dorsal) aspect of the rectum, to which a dermoid cyst, containing skin, hair, and teeth, is attached. (From the Middlesex Hospital Museum.

Dermoid *tumours* originating in the neurenteric canal present themselves as pedunculated tumours swinging from the inner wall of the rectum, sometimes as high as the sigmoid flexure; they may be attached to within two inches of the anus. The most typical examples known to me are those described by Dr. H. Port* and Mr. Clutton.† Dr. Port's case is represented on page 299, Fig. 4.

An abundant crop of long hair seems to be a common character of these rectal dermoid tumours. In several cases recorded incidentally in periodical literature, hairs more than two feet in length were found, and in some instances these so annoyed the patients that it was necessary to cut them on account of the inconvenience they occasioned during defæcation. Possibly the irritation of fæces may in part account for the abnormal length of the hair.

In Dr. Port's case the tumour measured 2½ inches in length, 2 inches in width, and 1½ inches in thickness. It is made up of fat, fibrous tissue covered with an investment of skin, provided with papillæ, hair follicles and sebaceous glands. One tooth was also found.

In the report of this case Dr. Port refers to a case published by Dr. Danzel‡ which occurred in a woman aged 25 years. This tumour coincided in structure with the tumour just considered, but with this addition: The microscope proved that brain substance, inclosed in a bony capsule, formed part of the tumour. This last fact is of very great interest for it serves to strengthen the opinion that these tumours owe their origin to the neurenteric passage. Of course the evidence on such questions as these must be always more or less circumstantial, but recently a very valuable example of abnormal development of the caudal end of the notochord has been published by Ryder.§

Fig. 11. A longitudinal section through the caudal region of a seventh-day chick. Ch. Notochord. N. Spinal cord. Nc. Neurenteric canal. P. Proctodaeum. I. Intestines.

The condition is represented in Fig. 11. In the drawing which was prepared from longitudinal sections of a seven day's chick, it will be seen that the notochord bifurcates posteriorly into a dorsal and vent-

*Path. Soc. Trans., Vol. XXXI., p. 307.
†*Ibid.*, XXXVII., p.
‡Langenbech's *Arch.*, 1874.
§American Naturalist, Vol. XX., p. 392, 1886.

ral portion. A bridge of tissue connects the dorsal spur with the spinal cord. Immediately above the bridge of tissue a vesicle is seen which must be regarded as a persistent portion of the neurenteric canal, which has become dilated. It is easy to see that with a persistent vesicle, abnormal arrangement of the notochord, and disturbed relation of the mesoblastic tissue in the immediate neighborhood, that had the chick lived, a sacral teratoma would probably have resulted.

3. *Lingual Dermoids.*—These form an instructive group and are interesting for they probably occur more frequently here than in any other region. There exist abundant reasons for believing that many cysts connected with the floor of the mouth and described as ranulæ and sebaceous cysts of the tongue were really dermoids, and many of the later specimens have been described as such.

Dermoids in the floor of the mouth may originate in three ways :—1. As sequestrations of epiblast in the middle line. 2. By dilatation of an unobliterated lingual duct. 3. From persistent branchial spaces.

The two latter forms will be dealt with together, for they are intimately associated. The first group have been already described in connection with sequestration cysts, and there it was mentioned that a duct was formed in the posterior part of the tongue as a result of the incomplete union of the two lateral halves of that organ.

4. *Branchial spaces.*—Dermoids in connection with these clefts are occasionally seen. The general relations and configuration of these clefts are too well known to need description. Dermoid cysts arising in unobliterated portions of these familiar fissures occur as a rule in the line of the sterno-mastoid muscle in various situations. They may be on a level with the thyroid cartilage or as low as the clavicle. In rarer cases they may extend into the parotid region or arise in connection with the pinna. It is significant that they lie in close relation with the anterior border of this muscle and in some instances extend deeply so as to gain an attachment to the sheath of the carotid vessels. As far as can be gathered from a large collection of published reports of these cases, they do not differ structurally from lingual dermoids. They possess a cutaneous lining, from which sprout hairs, furnished with sebaceous glands, the cyst-contents being loose hair, sebum, fat and cholesterin.

5. *Ovarian Dermoids.*—Dermoid cysts occur with extreme frequency in the ovary, indeed it has been stated that two-thirds of the total number of this form of tumour are found in connection with this organ. It is by no means uncommon to find a dermoid in each ovary as frequently as four times in sixteen cases. Notwithstanding the fact that they are so common in this situation, I have deferred their consideration to the end of this section, because it was essential to show the intimate connection between dermoid cysts in certain situations and obsolete canals. An endeavor will be made here to show that by far the larger number of ovarian dermoids arise in such canals. Of all organs in the body not one is so intimately associated, during its development, with so many tubular passages which eventually become obsolete as the ovary. All these canals are lined with a definite epithelium which is in all probability derived from hypoblast, possibly epiblast ; many of them open in the free surface of the body cavity, as rudimentary peritoneal funnels and in a situation famous for its high degree of potentiality—the genital ridge. Others of the ducts are closely associated with the mesonephros, and lastly we have the mesonephritic duct (Wolffian) in close relation with the surface epiblast. Now, it is a very remarkable fact that dermoid cysts of the testes are very rare, and this rarity is increased if care be taken to exclude dermoids arising as sequestration cysts in the middle line of the scrotum. This receives explanation from the fact that the ducts which become obsolete, or give rise to dermoids in the ovary, are in the testes functional excretory conduits.

It is also necessary in considering the ætiology of ovarian dermoids to draw a distinction between these cysts and those conglomerations of fœtal tissues which constitute the abnormality known as parasitic fœtuses, for they may occur in this situation as in any other region of the body.

Dermoids of the ovaries present much variety in structure. In the first place the number of hairs in a cyst may be so few as to be easily counted, or they may be innumerable. In length they vary from an inch to one or even two feet. The glands supplying these hairs are liable to considerable variation. In some instances they may be as large and conspicuous as in sections of the hairy scalp, or so small and scanty that perhaps a part of one or at most two appear in a single section. In others the glands are as large as those found in the skin of the nose, the dimensions of the gland being out of all proportion to the size of the hair,

Fig. 12. A dermoid cyst of the ovary with a mammary gland attached to its wall. (After Dr. von Velits.)

and furnishing an appearance on section as though the hair were loosely lodged in the duct of the gland, rather than the gland being an appendage of the hair.

The most extraordinary condition to which glandular formations attain in these singular tumours is, when a mamma, furnished with an areola and nipple is found in the cyst, and the development is complete, for on section the mass is found to present the structural characters of a mammary gland. An example of this form of cyst is shown in Fig. 12. The case was reported by Dr. Desiderius von Velits, of Budapest. Not merely did it resemble a mamma in structure, but milk and colostrum globules could be squeezed from it.

Lannelongue states that Haffter found in the parietes of an ovarian dermoid a glandular mass, resembling a rudimentary mamma, and refers to a case recorded by De Sinety, in which an ovarian cyst contained a glandular structure resembling a mammary gland (*Traite pract. de gynecologie*, 2e edit, Paris, 1884, p. 749).

Cysts containing structures of this character are very rare. As far as I am aware von Velits'* case is the most extreme instance known. The case serves to support the view that the mamma is an excessively developed dermal gland.

With regard to teeth, the cyst may contain none, or be

Fig. 13. A piece of bone supporting teeth; from an ovarian dermoid cyst.

Fig. 14. Section of an ovarian dermoid cyst. Each loculus contained sebaceous matter and cholesterin. From the walls sprouted hair and the cyst contained upwards of 200 teeth.

studded with teeth of various sizes and shapes to the number of one, two, or even three hundred. When small in number the teeth may grow from definite bony masses, which may faintly bear some resemblance to an upper maxilla. The teeth resemble the milk dentition, and some

1 Virchow's Archivs, Bd. 107, S. 505.

observers have described germs as of permanent teeth growing in relation with them. As to this I have failed to satisfy myself. The teeth present the usual disposition of enamel. dentine, cementum and pulp, and it is a fact somewhat remarkable that caries with extreme rarity attacks these teeth. Secondary dentine may, however, be found in the pulp chamber. The walls of ovarian dermoids occasionally calcify.

In size an ovarian dermoid may vary from that of a nut to an enormous mass reaching from the pelvis to the umbilicus, and on several occasions a suppurating ovarian dermoid has opened or been opened by the scalpel at the navel. The most remarkable condition, which, so far as I know, has only been noticed in connection with ovarian dermoids, is the distribution of a number of smaller cysts among the omentum, mesentery and small intestines. The most carefully reported case of this nature is by Mr. Moore, in the *Trans. Path. Soc.*, Vol. XVIII. Mr. Moore, however, points out that the majority of these secondary cysts had long pedicles of attachment to the main cyst. This may help to explain a curious situation occasionally attained by them, which we will now proceed to consider. Among dermoids originating in obsolete canals, such as we have been considering, must be included many of those abdominal dermoids described as non-ovarian. The cysts of this group are of great clinical importance as well as being interesting pathologically.

Mr. Alban Doran,* in a careful paper on this matter, adduces excellent arguments in support of the view that many dermoid cysts of the abdomen that have been described as non-ovarian, are really ovarian cysts that have become separated from their pedicles, and this is especially the case with regard to cysts of the great omentum. It is equally certain, as Doran remarks, that all abdominal dermoids are not of this nature ; as, for example, those very rare instances of abdominal dermoids occurring in male subjects.

---

* Notes on so-called Non-ovarian Dermoid Abdominal Tumours." *Med. Chir. Trans.*, Vol. LXVIII., p. 235.

Certainly dermoids situated in the space between the rectum and bladder in men and in the fossa of Douglas in women, admit, as has already been stated, of another interpretation.

The greatest argument adduced by Doran in support of his view is a case reported by him* which was under the care of Mr. Thornton. The tumour had existed in the abdomen for seven years, during which period the patient gave birth to four children. At the operation a dermoid cyst was found closely adherent to the omentum. The left ovary was absent, a short, firm tag of tissue hanging from the omentum close to the Fallopian tube.

Recently it has been my good fortune to have the opportunity of examining a very interesting case of detachment of an ovarian dermoid, which was exhibited to the British Gynæcological Society (1887) by Grigg Smith, and of which I furnished an independent report. In this case the cyst was a compound of a simple ovarian (oophoritic) cyst and a dermoid. It was adherent to the omentum, but a frail, thin band of tissue connected it with the right angle of the uterus. An examination showed that the tumour consisted of three inches of the Fallopian tube, the ovary was full of small, abnormally distended follicles, and surmounted by a cyst as large as an ordinary melon. In the middle of this ovary was a dermoid of the size of a tangerine orange, full of oily material; from the walls sprouted a few stunted hairs, furnished with very large-branched sebaceous glands.

The thin ligament which connected the tumour with the uterus was the attenuated portion of the Fallopian tube of the thinness of an ordinary silk ligature.

The examination of this tumour furnished one fact of peculiar interest. Although the tumour was of large size the only point at which omental adhesions existed was that part of the tumor composed of the dermoid. It is a fact well recognized that dermoids are especially prone to contract adhesions to surrounding parts, and many in-

---

* Observations on Tumours of the Ovary, &c.

stances are on record in which they have suppurated. In some cases the pus escapes externally and a spontaneous cure is effected or bursting internally terminate the life of the patient. In rare cases malignant growths may spring up in dermoids, and Doran is of opinion that many of the cartilage knobs, osseous masses, etc., in dermoids may be regarded as enchondromata, osteomata, etc.—in fact they may be considered as tumours of a tumour.

The last class of abdominal dermoids which remain for our consideration are those which occupy the space between the rectum and bladder. A study of this class of dermoids is of some value, for it seems to me that it is this variety which has given colour to the opinion that they are due to dropped ova, that is, impregnated ova which miss the Fallopian tube. This absurd view is refuted by the fact that they occur in males and a characteristic example is recorded by Dr. Ord.* The fact, too, that they may be found in man also negatives the possibility that they are ovarian dermoids which have been detached.

Dermoids in this situation I believe to arise in the neurenteric passage and have no connection either with ovary, testicle or dropped ova.

We must not dismiss the consideration of ovarian dermoids without drawing attention to the relation they have with those rare cases in which hairs are either passed with the urine, or as the nucleus of a vesical calculus.

Hairs occurring in the bladder may be derived from two sources. (1) Their introduction from without, accidental or otherwise. (2) An ovarian dermoid may rupture into the bladder and its contents, hair, cholesterin and sebaceous matter voided through the rectum. A full consideration of recorded cases of this event leads me to believe that in most cases this accident is determined in consequence of inflammation and suppuration of the cyst. One of the best descriptions known to me of the rupture of a dermoid with discharge of the contents through the urethra with an account of its distressing concomittants is by Dr. Robert Lee.†

* Med. Chir. Trans., Vol. LXIII., p. 1. The paper contains several references.
† Med. Chir. Trans., Vol. XLIII., p. 93.

*Conclusion.*—In the main this article has been one long argument to prove that dermoid cysts and tumours have their origin in detached portions of epiblast, and in epithelial-lined tubes which should, in the ordinary course of development, become obsolete.

It also insists on the importance of distinguishing between cysts originating in this manner, and those heterogeneous masses which represent suppressed or parasitic fœtuses.

Further, those congenital neoplasms containing striated muscle-fibre connected with the kidney and spinal cord do not belong to this category. Finally, the study of dermoids lends, to my mind, the strongest possible support to Cohnheim's view—that many neoplasms originate in detached masses of embryonic tissues.

Labour in the direction of references has been spared me by the timely appearance of Lannelongue's *Kystes Congenitaux*, 1886, which contains an enormous assembly of cases scattered in classical and periodical literature, all of which are carefully and judiciously classified. My indebtedness to this painstaking author is very considerable. The Pathological Transactions have served as a mine, in which I have freely delved, and the memoirs of Cusset, Wagstaffe and Doran have been to me of especial value.

Virchow's invaluable Archivs can only be compared to the Path. Transactions, which contain many instructive and carefully reported specimens of these interesting abnormal conditions.

Other sources of information are fully acknowledged in numerous foot-notes.

## Figures and Tables

**Fig. 1. f1:**
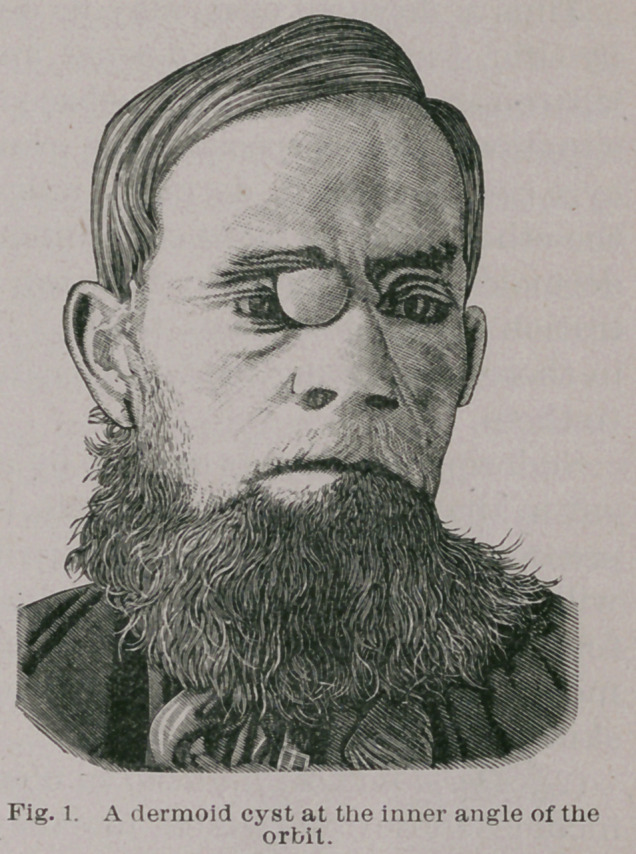


**Fig. 2. f2:**
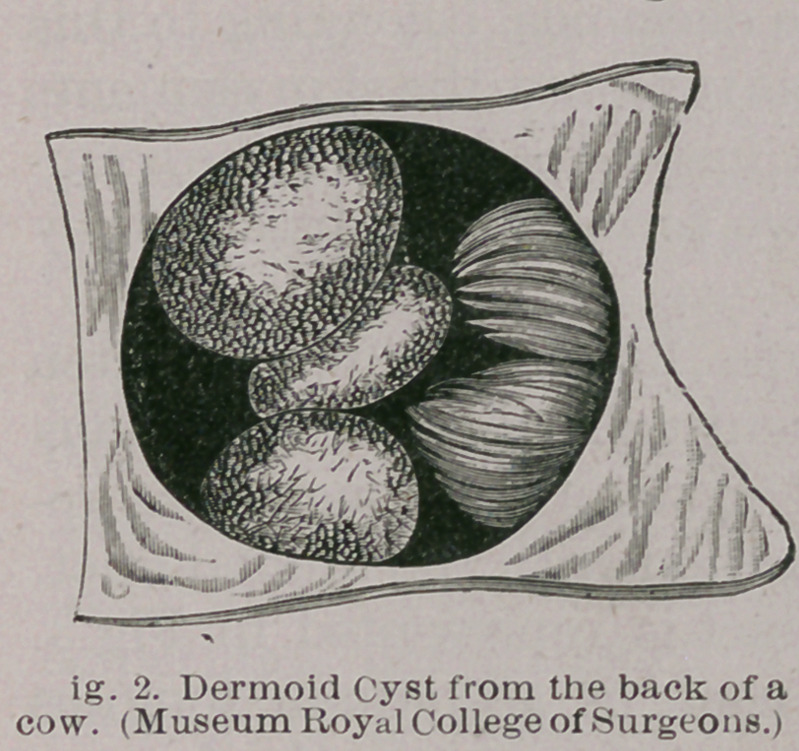


**Fig. 3. f3:**
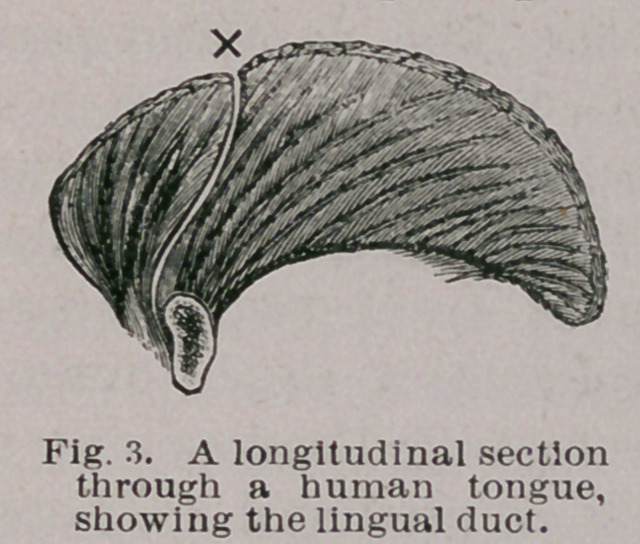


**Fig. 4. f4:**
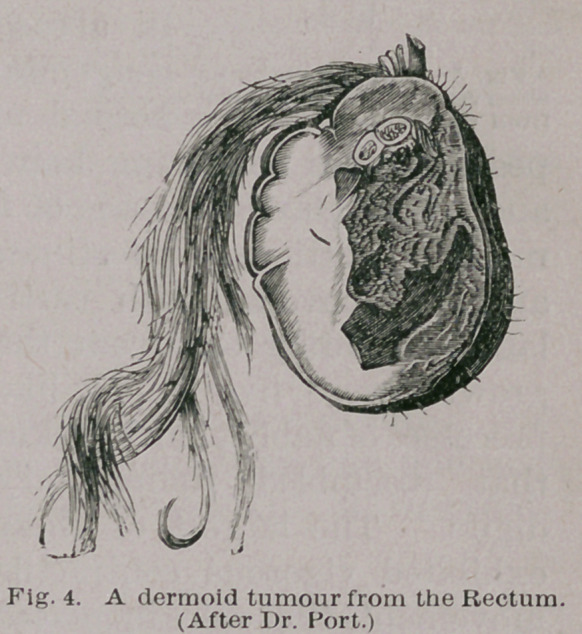


**Fig. 5. f5:**
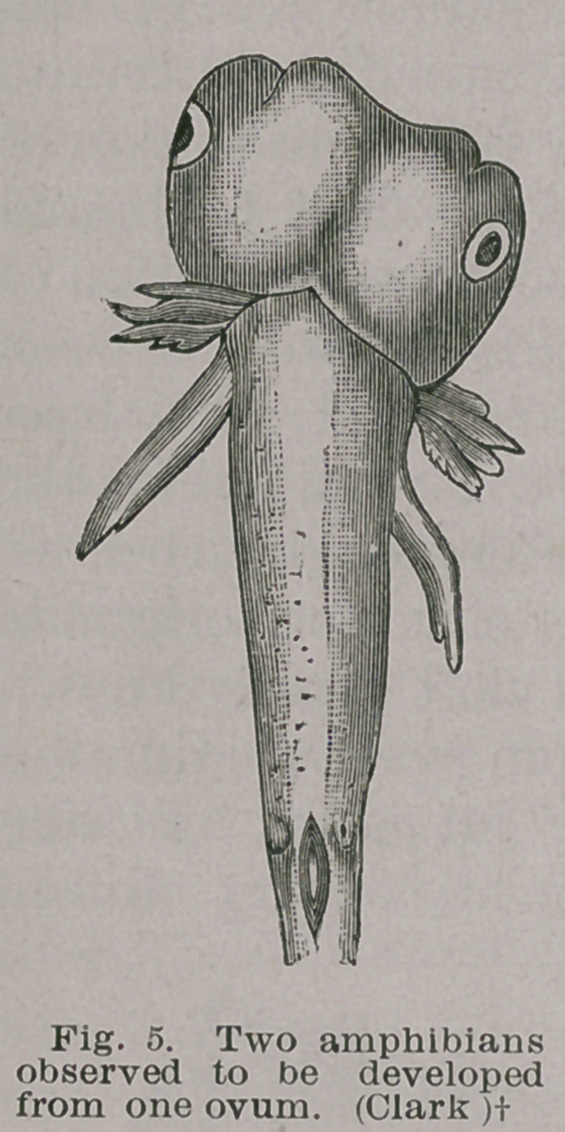


**Fig. 6. f6:**
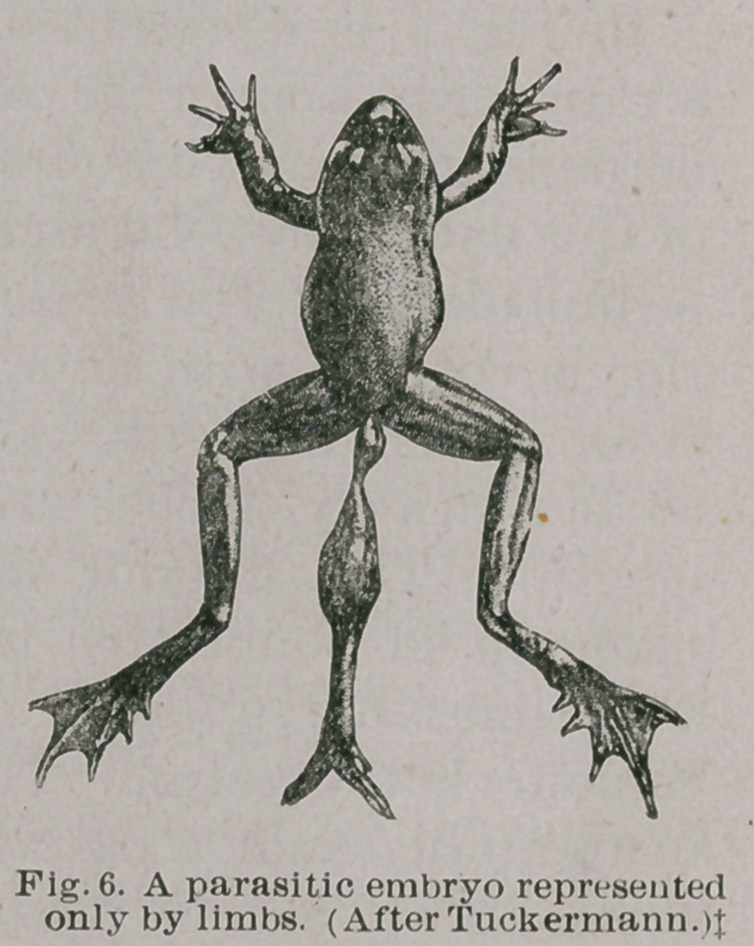


**Fig. 7. f7:**
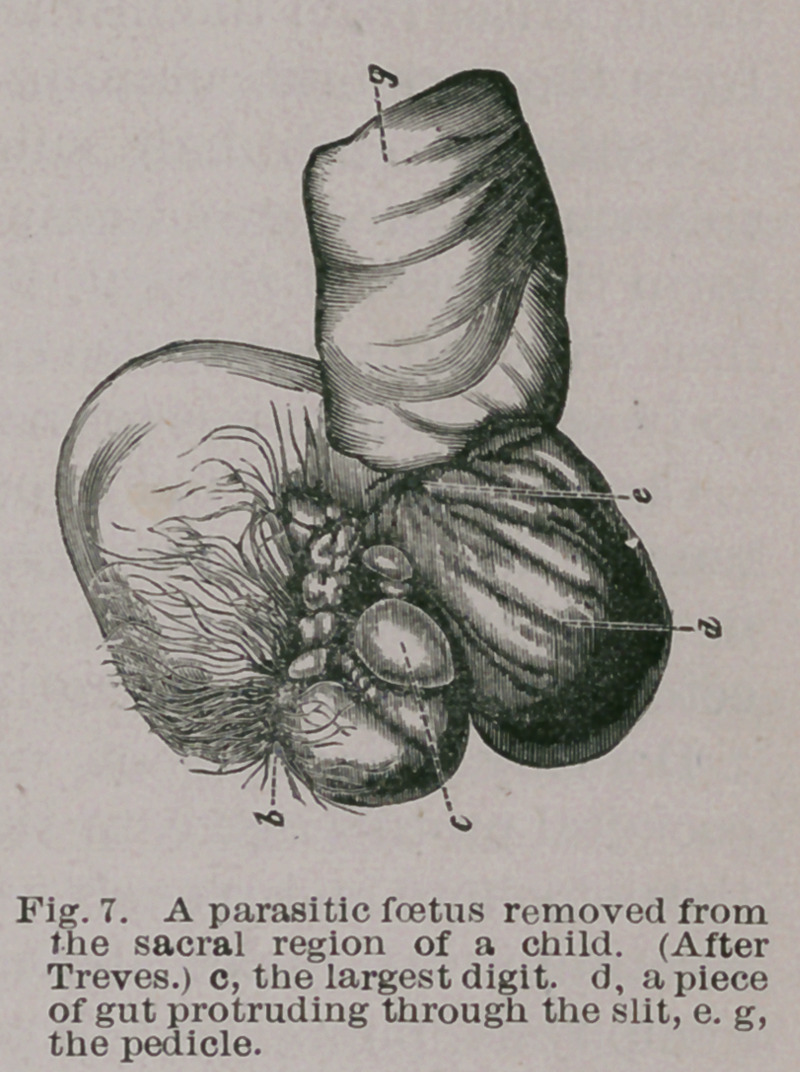


**Fig. 8. f8:**
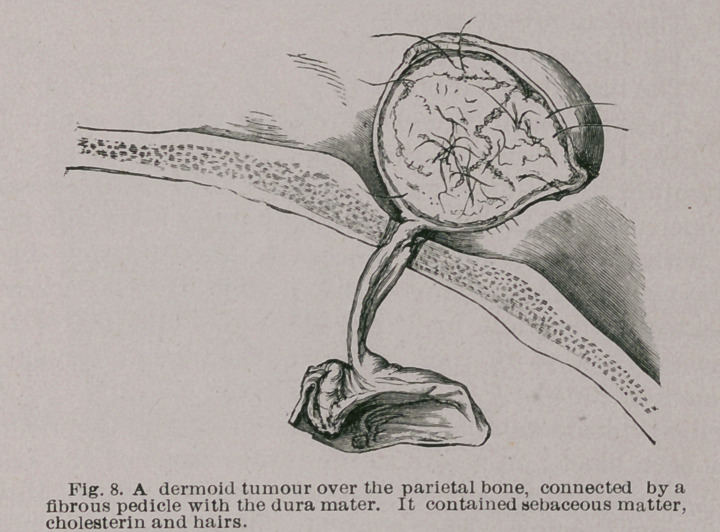


**Fig. 9. f9:**
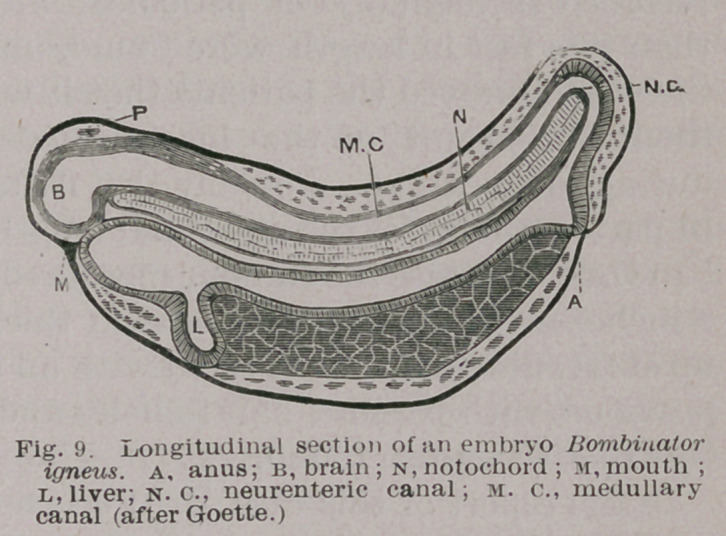


**Fig. 10. f10:**
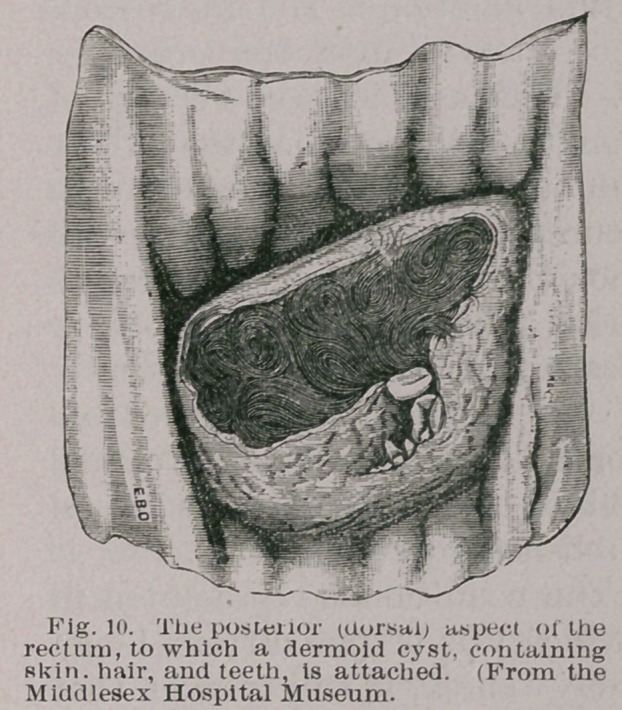


**Fig. 11. f11:**
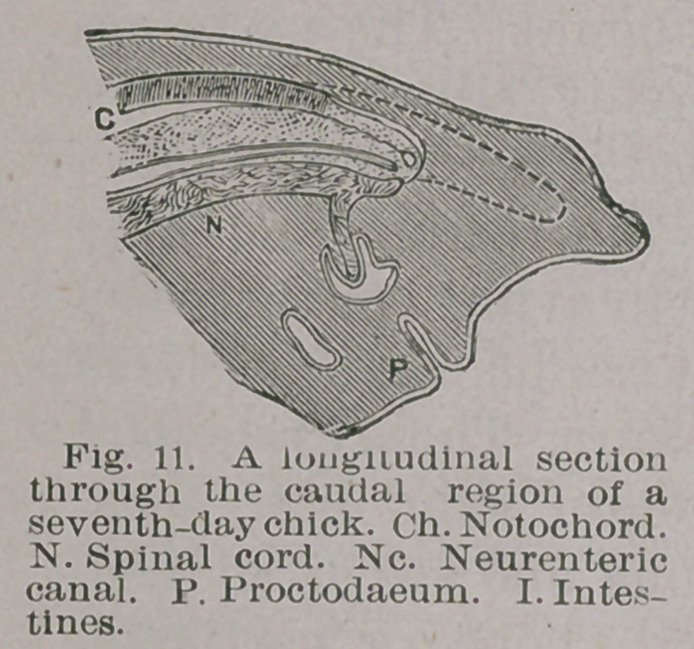


**Fig. 12. f12:**
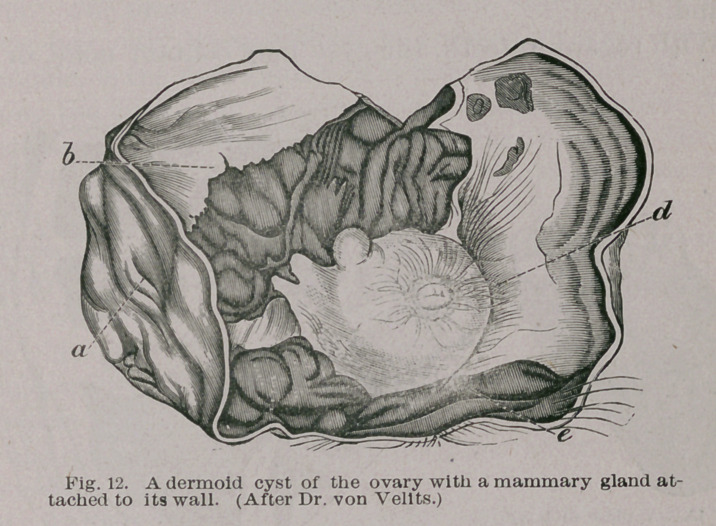


**Fig. 13. f13:**
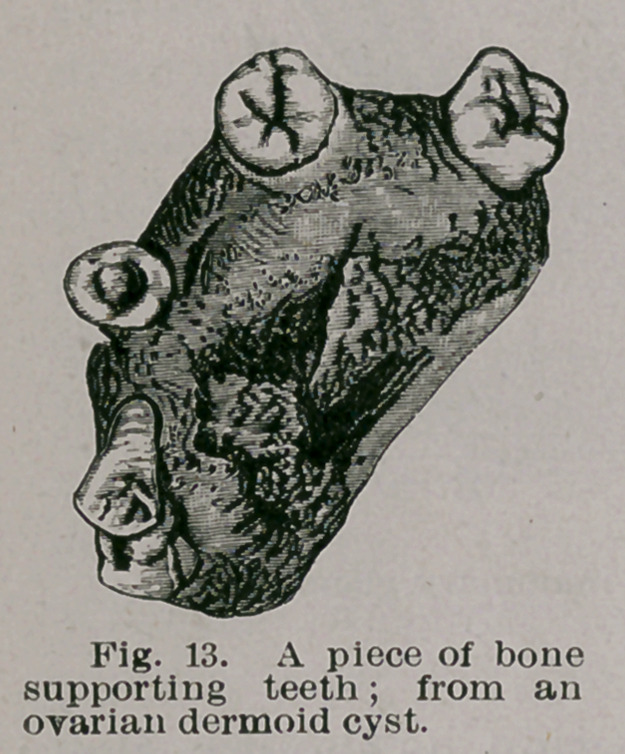


**Fig. 14. f14:**